# Flow Cytometry Pulse Width Data Enables Rapid and Sensitive Estimation of Biomass Dry Weight in the Microalgae *Chlamydomonas reinhardtii* and *Chlorella vulgaris*

**DOI:** 10.1371/journal.pone.0097269

**Published:** 2014-05-15

**Authors:** Maurizio Chioccioli, Ben Hankamer, Ian L. Ross

**Affiliations:** Institute for Molecular Biosciences (IMB), The University of Queensland, Brisbane, Queensland, Australia; Center for Nanosciences and Nanotechnology, Mexico

## Abstract

Dry weight biomass is an important parameter in algaculture. Direct measurement requires weighing milligram quantities of dried biomass, which is problematic for small volume systems containing few cells, such as laboratory studies and high throughput assays in microwell plates. In these cases indirect methods must be used, inducing measurement artefacts which vary in severity with the cell type and conditions employed. Here, we utilise flow cytometry pulse width data for the estimation of cell density and biomass, using *Chlorella vulgaris* and *Chlamydomonas reinhardtii* as model algae and compare it to optical density methods. Measurement of cell concentration by flow cytometry was shown to be more sensitive than optical density at 750 nm (OD_750_) for monitoring culture growth. However, neither cell concentration nor optical density correlates well to biomass when growth conditions vary. Compared to the growth of *C. vulgaris* in TAP (tris-acetate-phosphate) medium, cells grown in TAP + glucose displayed a slowed cell division rate and a 2-fold increased dry biomass accumulation compared to growth without glucose. This was accompanied by increased cellular volume. Laser scattering characteristics during flow cytometry were used to estimate cell diameters and it was shown that an empirical but nonlinear relationship could be shown between flow cytometric pulse width and dry weight biomass per cell. This relationship could be linearised by the use of hypertonic conditions (1 M NaCl) to dehydrate the cells, as shown by density gradient centrifugation. Flow cytometry for biomass estimation is easy to perform, sensitive and offers more comprehensive information than optical density measurements. In addition, periodic flow cytometry measurements can be used to calibrate OD_750_ measurements for both convenience and accuracy. This approach is particularly useful for small samples and where cellular characteristics, especially cell size, are expected to vary during growth.

## Background

Biomass dry weight reflects photosynthetic carbon fixation. Its accurate estimation is a longstanding problem in oceanography and freshwater biology and is a key economic determinant for algal biofuel biotechnology [1], as well as many industries which utilise microbial culture systems [2].

The standard technique for measuring biomass dry weight is direct weighing [3]. However, the accuracy of weighing equipment usually restricts the sample size to relatively large (mg) amounts of material. Furthermore, since direct weighting is time consuming, this technique is not suitable for applications requiring feedback control.

The use of high-throughput research techniques, such as screening assays in microwell plates, requires continuous or semicontinuous monitoring of biomass yield in a multitude of small volume samples grown under thousands of different conditions. These small volumes necessitate an indirect way to estimate biomass dry weight. Many options exist, each with specific advantages and limitations.

Particle analysis instruments have long been used for algal cultures and utilise an electrochemical measurement of particle size (Coulter principle) or an optical measure of particle Brownian motion (dynamic light scattering). These approaches use the measured particle diameter to automatically calculate the included wet biomass volume on the assumption that the particle is spherical. Conversion from buoyant mass or biovolume to dry weight is problematic, as the dry content of cells is highly variable both within and between populations [4] and so a constant average density cannot be assumed.

For routine laboratory algaculture, optical density at 750 nm (OD_750_) is widely used to monitor algal growth, being inexpensive and reliable. Measurement at 750 nm avoids the absorption of light by cellular pigments (chlorophyll and carotenoids) and is treated as a pure light scattering measurement. The major drawback with OD_750_ measurements is that light scattering is an aggregate variable of cell size, density, opacity and granularity which is difficult to deconvolute, and may also be distorted by the presence of bacteria and inorganic solids. As long as all cultures being assayed share the same average cellular characteristics, extrapolation from OD_750_ to biomass dry weight is straightforward and simply requires an empirical calibration for each species. When cellular characteristics change during growth, however, the relationship between OD_750_ and biomass also changes. The resulting inaccuracy is sometimes inconsequential, but becomes important when one of the primary variables of interest is the biomass yield itself. Furthermore, OD_750_ measurements convey little information about the cell concentration or the size distribution within the population, both of which are of interest because they reflect cellular physiology.

Photomicrography followed by image analysis offers the opportunity to directly measure a range of cellular characteristics in a culture. If cell density changes little during growth, cell diameters can be used to estimate biovolume and wet mass. Image analysis requires the automation of numerous steps to avoid laborious manual processing [5]–[7]. Specialised commercial devices are available for this, but again, the estimation of dry mass from biovolume is the major obstacle.

Flow cytometry has long been used for tracking algal populations and estimating biomass especially in environmental samples [8], [9]. Typically, this technique is used for large scale biomass estimation in natural water bodies and usually relies upon the use of empirically derived, published values relating average carbon content to forward scatter pulse area (FSC-A), chlorophyll fluorescence or DNA content for each given species [10], [11]. Thus it is most commonly applied to the approximation of total organic carbon contributed by low cell abundances within a complex ecological system (e.g. ocean strata) rather than precise analysis of biomass in a high density controlled culture. In contrast to forward scatter, side scatter pulse area data (SSC-A) is generally taken to reflect "granularity"; cells with more complex internal structure scatter light more in all directions, which is detected by the side scatter detectors.

Flow cytometry offers a rapid and accurate analysis of cell concentration and is widely available and frequently used in algal research. It not only provides information about cell size, but has the potential to simultaneously analyse many other biological properties including DNA, protein, pigment and lipid content, as well as the amounts of specific biomolecules through the use of fluorescent labelling. This flexibility is one reason for the popularity of flow cytometry in biomedicine and is an attractive feature for the analysis of algal species. Sample measurement is also quick and easy once protocols have been established, especially when fixed cells are used for batch processing.

Our laboratory has been utilising high throughput assays in microwell plates to explore a variety of algal culture conditions. Here we present data identifying conditions where OD_750_ measurements used to define algal biomass dry weight become unreliable, and show how flow cytometry can be used as a complementary technique. In contrast to pulse area data (either FSC-A or SSC-A), pulse width data is a rarely used parameter, mainly employed to discriminate between single-cell and twin-cell events. Here, a relationship between flow cytometric pulse width data and biomass dry weight is identified, enabling the estimation of dry weight from side scatter data given appropriate calibration for each species. The relative advantages of each technique are illustrated as well as the benefits of using them together.

## Methods

### Cells and growth conditions

Flask cultures of *Chlorella vulgaris* (CCAP 211/11B, Culture Collection of Algae and Protozoa, UK) and *Chlamydomonas reinhardtii* strain 137c (Chlamydomonas Center Collection www.chlamy.org, DUKE University, USA) were photoheterotrophically grown in Tris-Acetate-Phosphate (TAP) medium [12] in 25 mL Erlenmeyer flasks at 22°C under continuous illumination at 50 µE m^−2^ s^−1^ with cool-white fluorescent light (Philips Lifemax TLD58W/840, Philips Australia). Flask cultures were shaken on an orbital shaker at 100 rpm to ensure uniform illumination and to prevent cell settling. Starter cultures (10 mL) prepared by inoculation of TAP from a plate or previous liquid culture and grown to log phase or early stationary phase were sub-cultured into 100 mL fresh TAP.

Prior to initiating each long term growth experiment, synchronization of the cultures was performed [13] by imposing a regime of alternating 12h light and dark periods for 3 days with 60 µE m^−2^ sec^−1^ in the illuminated phase, and by diluting the culture to 2×10^6^ cells mL^−1^ immediately prior to the onset of the light period. After 3 such cycles, the culture was considered synchronized, verified in initial experiments using flow cytometry (data not shown). Once the experiment began, lighting was continuous.

The growth phase of the culture was tracked to the mid-logarithmic phase by diluting a sample 10-fold into TAP and measuring the optical density at 750 nm (OD_750_) using a Biorad SmartSpec spectrophotometer. OD_750_ measurements for samples in microplates were taken using a BioTek Powerwave XS plate reader (BioTek USA), subtracting the average value of OD_750_ taken from at least 5 blank wells containing the same volume of TAP.

Microplate experiments were carried out by diluting starter cultures into either TAP, Tris-Phosphate (TP; i.e. TAP without acetate, but at the same pH) or TP with 100 mM glucose. All base media were sterilised by autoclaving except glucose stocks which were filter sterilised. The OD_750_ measured in the spectrophotometer (1 cm path length) was approximately 6 times the uncorrected OD_750_ measured in the microwell plate (assuming a 150 µL volume). Optical densities stated in the text or figures (without qualification) refer to the values as measured in the plate reader.

Estimation of µ (specific growth rate) [14] from OD_750_ or cell concentration data was carried out using an exponential least squares fit (GraphPad Prism; model y = y_0_ *e* ^µt^ where y_0_ is the initial value of y, t is time and µ is the rate constant i.e. specific growth rate) with manual inspection of semilog plots to verify fitting accuracy.

### Flow cytometry

Cytometric analysis was carried out using a BD FACS Canto II Flow Cytometer (BD, San Jose, USA) fitted with a high throughput system (HTS) to analyse multiwell plate samples. Cell sorting was carried out using a BD FACS Aria cytometer. Forward scatter (FSC) and side scatter (SSC) measurements were taken using the 445 nm blue laser and routine fluorescent gating of algal cells was carried out using the 670 nm long pass filter (670LP) which detects chlorophyll fluorescence to define a population which was consistent with the size of algal cells (high in forward scatter area; FCS-A) and high in chlorophyll fluorescence area (high 670LP-A). This excludes any cell debris, bleached cells or bacteria. Normally, gated algal populations were the dominant type of event detected.

For multiwell analyses, flow cytometry (FC) was performed with the reading time as short as possible to maximise sample throughput: Typical FC parameter settings were: flow rate 1.0 µL s^−1^, sample volume 10–30 µL, mixing volume 30 µL, mixing speed 180 µL s^−1^, no. of mixes 5, washing volume 200 µL. Typical photomultiplier voltage gain parameters were: FSC 350 V (log scale), SSC 300 V (log scale), 670 nm long pass filter 300V (log scale). Each FC gate was set around microalgae populations to exclude any non-fluorescent particles. FACS Diva software (BD, San Jose, USA) was used for data acquisition and analysis.

Absolute microalgae cell counts were obtained by adding an internal microsphere cell counting standard (Count Bright; Invitrogen CA) to the flow cytometric sample (single platform testing). A 1∶9 dilution of Count Bright suspension was added to the microalgae culture. Using a FSC-A vs. SSC-A plot to separately gate cell events vs bead events, the ratio of bead events to cell events (together with the known concentration of beads) was used to calculate the absolute cell concentration. This did not vary more than a few percent from simply counting the number of cell events by comparison to an external cell suspension with a known cell concentration based on haemocytometer counting.

### Estimation of cell diameter and biomass volume from pulse width flow cytometry data

The procedure of Hoffman [15] was used to calibrate side scatter pulse width data from cells against size calibration bead standards of either polystyrene (Spherotech Inc.) or silicon dioxide (Micro Particles GmbH). Briefly, a single bead size was used to determine the range of amplifier gains where the pulse width was independent of peak height or peak area. The remainder of the calibration was performed within this range. The side scatter pulse width (SSC-W) of the bead standards was then plotted against bead diameter and fitted with a nonlinear curve according to Sharpless and Melamed [16]. This calibration curve was then used to transform the median SSC pulse width data from a given cell population into a median cell diameter. For hydrated biomass volume estimation, the SSC-W histogram was divided into 5–10 bins. The median cell diameter in each bin was estimated and used to calculate the median cell volume for the bin, and the cell frequency per bin (estimated from counting standard beads) was used to estimate the net hydrated volume per bin. Finally the net volumes of each bin were summed to generate the total biomass volume.

### Gravimetric dry weight estimation

Biomass production in grams dry weight (g _dw_) L^−1^ was estimated for flask cultures by collecting 3×10 ml at lag phase (zero time), log phase (after ∼10 hours of growth) and stationary phase (approx. 24–27 hours). The culture was centrifuged (1500 x g, 5 min) in sterile 15 mL Falcon tubes (Becton Dickinson). The pellet was then gently washed with 2 mL of sterilised MilliQ water to remove culture medium salts. The sample was again pelleted (1500 x g, 5 min), the supernatant was carefully removed and 1.5 mL of sterilized water used in several aliquots to resuspend the pellet into pre-weighed 1.5 mL Eppendorf tubes. Cells were again pelleted at 1500 x g for 5 min, and the supernatant discarded. Tubes were dried overnight at 1500 rpm 30 mbar 25°C in a vacuum centrifuge (Christ Alpha-RVC Speedivac, Christ Osterode am Harz, Germany) until the weight remained at a constant minimum. Alternatively, samples were oven-dried at 80°C until a constant minimum weight had been achieved. Tubes were weighed on a 6 figure precision balance (Shimadzu AUW220D) to estimate the weight of the dried biomass. Ash free dry weights were not calculated since for these strains ash contents are below 5%[17]. Strains with higher ash contents (especially diatoms, marine strains) would require the ash content to be taken into account.

### Percoll density gradient centrifugation

Buoyant cell density was estimated using Percoll density gradient centrifugation. Sterile Percoll (3 mL; GE Healthcare) was added to 300 µL liquid culture and mixed prior to centrifugation. A control tube was made using TAP in place of algal culture and 40 µL coloured marker beads mixture (Pharmacia). Bead densities were: Violet 1.138, Red 1.12, Green 1.102, Red 1.084 and Blue 1.019. Gradient tubes were then subjected to density gradient centrifugation at 40000 rpm for 1h using a TL100 benchtop ultracentrifuge (Beckmann) with a TL100.3 fixed angle rotor.

### Photomicrography

Photomicrographs of algal cells were taken using an inverted Nikon microscope (Nikon Ti-U Inverted Fluorescence Microscope) and the cell diameters estimated both manually and using IMARIS image analysis software.

## Results

### Cellular characteristics of microalgae can change during growth

In wild type *Chlamydomonas reinhardtii*, cell size varies predictably within the growth cycle. Cell size varies with strain but Umen and Goodenough [18] reported an average cell size around 75–150 µm^3^.

**Figure 1:**
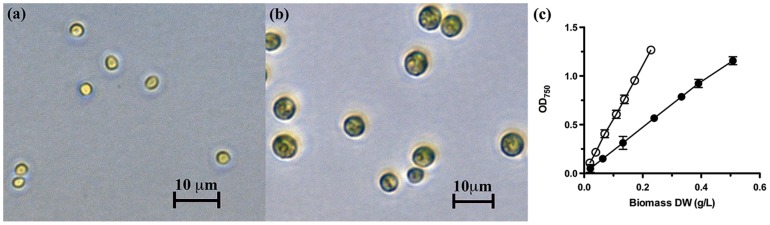
Cell size affects OD_750_ measurements. (a) Cells of *Chlorella vulgaris* grown photoautotrophically in unsupplemented TP have a smaller average diameter than (b) cells grown to stationary phase in Tris-Phosphate (TP) medium containing 100 mM glucose. (c) Changes in cell morphology affect the relationship between OD_750_ and biomass dry weight, yielding different calibration factors (line slope) for *C. vulgaris* in TP (○) and in TP + 100 mM glucose (•). Error bars are standard error of the mean (SEM; n = 3).

In contrast, the size and shape of *Chlorella vulgaris* is highly dependent on the culture conditions (Figure 1a, b). During the early logarithmic growth phase, microscopic observation indicated that *C. vulgaris* cells did not vary greatly in diameter during growth on media consisting of either Tris-phosphate (TP), Tris-acetate-phosphate (TAP) or TP + glucose, as cell division was rapid and most cells were small, newly divided daughter cells. As stationary phase approached, the rate of cell division slowed for cells grown on glucose (as evidenced by lower cell concentrations relative to the other conditions), and the cells began to increase in size to around 6–8 µm diameter (∼110–270 µm^3^). As a result, calibration curves of OD_750_ vs. biomass have markedly different slopes (calibration factors; CF) for each population (Figure 1c).

Average cell size also alters during growth when cell replication is synchronised to a day-night cycle, as is usually the case in outdoor cultures. For species with a strong circadian rhythm, synchronisation can be maintained for several replication cycles even under continuous light. Robust biomass estimation methods need to be able to account for the effect of this circadian cycle.

Based on gravimetric analyses, when the OD_750_ = 1.0, the population grown exclusively in TP yield a biomass concentration of 0.18 g _dw_ L^−1^ whereas those in TP + glucose yield 0.43 g _dw_ L^−1^. These OD_750_ calibration factors refer to the same strain, simply grown under different conditions which result in different cell size distributions. Quite possibly more extreme conditions would lead to even more discrepant calibration factors. Without knowing the precise population size distribution of a particular culture, the relative contribution of different OD_750_ calibration factors cannot be estimated, and hence an accurate biomass cannot be calculated, though limits to biomass can be estimated. We therefore investigated whether flow cytometry might offer a more accurate means of biomass estimation.

### Comparative sensitivity of OD_750_ and flow cytometry (FC)

We initially tested the relative sensitivity of FC and OD_750_ for measuring cell density. To measure growth rates accurately in microwell plates, cell densities must initially be low (<10^6^ cells mL^−1^) so that true exponential growth occurs prior to nutrient limitation and self-shading effects. The greatest accuracy of measurement is therefore needed during early log phase where the growth rate is maximal. At such cell densities, and with a short effective path length, OD_750_ microplate reader measurements suffer from a low signal intensity, even compared to a conventional spectrophotometer with a 1 cm path length. As flow cytometry requires relatively few cells, it was initially trialled as a way to estimate growth rates with greater sensitivity than OD_750_.

**Figure 2:**
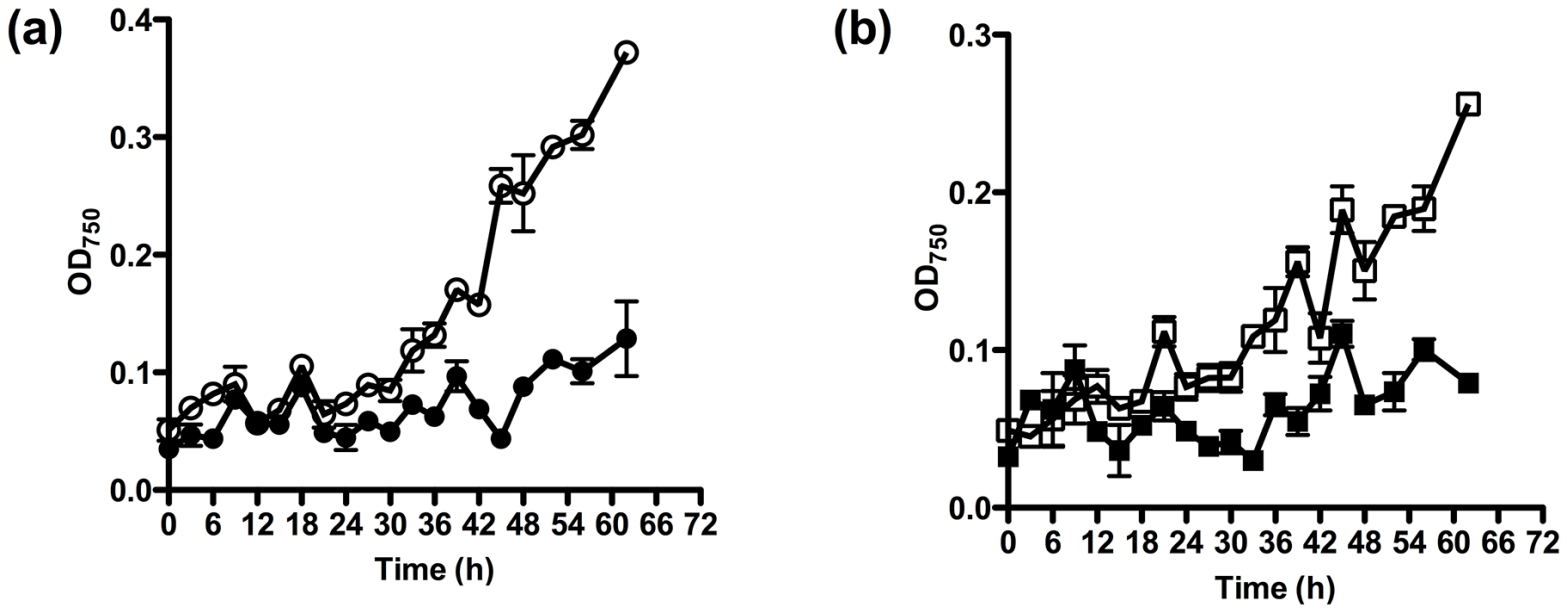
Growth curves monitored in 96 well plates by OD_750_ in a plate reader (effective path length ∼0.2 cm) at different initial cell densities. (a) *Chlamydomonas reinhardtii* in TAP at initial cell concentration of 10^6^ cells/mL (○) and at initial cell concentration of 2.5×10^5^ cells/mL (•). (b) *Chlorella vulgaris* in TAP at initial cell concentration of 10^6^ cells/mL (□) and at initial cell concentration of 2.5×10^5^ cells/mL (▪). Error bars are SEM (n = 3).

**Figure 3:**
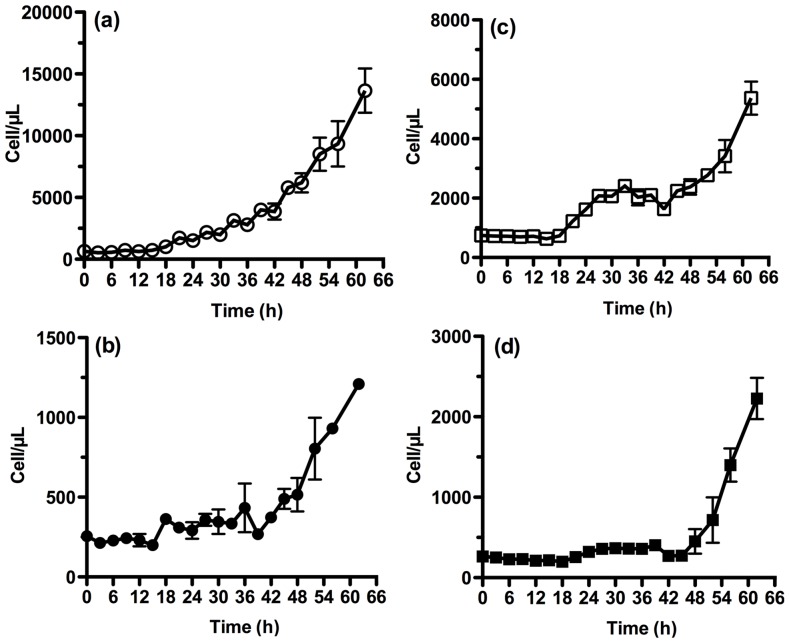
Growth curves monitored in 96 well plates, measuring cell concentration with flow cytometry. Cell concentration (cells/µL) was estimated from flow cytometry events, calibrated using CountBright Absolute Counting Beads (Invitrogen). Error bars are SEM (n = 3). (a) *Chlamydomonas reinhardtii* strain 137c at initial cell concentration of 10^6^ cells/mL (○). (b) *C. reinhardtii* strain 137c at initial cell concentration of 2.5×10^5^ cells/mL (•). (c) *Chlorella vulgaris* at initial cell concentration of 10^6^ cells/mL (□). (d) *C. vulgaris* at initial cell concentration of 2.5×10^5^ cells/mL (▪).

Parent cultures were synchronised to a 12h day-night cycle for 3 days and cell suspensions were then prepared at two concentrations; "concentrated" (∼1.0×10^6^ cells mL^−1^) and "dilute" (2.5×10^5^ cells mL^−1^) for both *Chlorella vulgaris* and *Chlamydomonas reinhardtii.* These were plated out in triplicate into microwell plates and were grown for 60 h under continuous light (100 µE m^2^ s^−1^) with shaking at 800 rpm, measuring microplate OD_750_ (Figure 2) and cell density by flow cytometry (Figure 3) every 3 hours. Calibration beads were included during flow cytometry to accurately estimate cell density.

Despite initial synchronisation, growth of the concentrated culture of *C. reinhardtii* proceeded smoothly and exponentially in continuous light, after an initial 12 hour lag period, as measured by flow cytometry (Figure 3a,b). Figure 2a shows that the OD_750_ data taken from the same cultures was also consistent with exponential growth, even though at these low cell concentrations, optical density data is less reliable. Flow cytometry enabled a reliable estimate of specific growth rate (µ = 0.05 h^−1^) to be obtained even at these low cell numbers, using a population growth model [14]. All cultures demonstrated a lag phase with effectively zero growth, clearly visible in log-transformed data (Figures S1 and S2). To estimate the exponential growth rate during the replicative cycle, data following the initial lag phase was fitted using an exponential least squares fit. Growth rates and doubling times are given in Table 1.

**Table 1:** Growth constant µ (h^−1^) estimated by OD_750_ or flow cytometry (FC).

	*Chlamydomonas reinhardtii*		*Chlorella vulgaris* †	
**Initial OD_750_**	µ (OD_750_)×10^3^ h^−1^	µ (FC)×10^3^ h^−1^	µ (OD_750_)×10^3^ h^−1^	µ (FC)×10^3^ h^−1^ †
**0.025**	24.6±4.4 (0.61)*	44.3±4.4 (0.77)	20.6±5.2 (0.46)	107.6±10.1 (0.94)
**0.1**	39.1±3.8 (0.92)	55.5±2.6 (0.95)	29.6±2.2 (0.85)	57.6±3.8 (0.94)

*+/− SEM with r^2^ value in parentheses.

† Although fitted with an exponential model, cell replication (seen by flow cytometry) is not truly exponential but reflects the number of daughter cells per replication cycle.

For *C. reinhardtii* the growth rates estimated from flow cytometry (∼0.04–0.05h^−1^) are similar to each other regardless of the initial OD_750_ of the culture, and also similar to the growth rates derived from OD_750_ data, except for the rate estimated from the culture with low initial OD_750_ (∼0.02 h^−1^) which can be attributed to the more variable data at low OD_750_ as seen by the low r^2^ value (0.61).

For *C. vulgaris*, rates estimated from OD_750_ measurements are lower than for flow cytometry because these two techniques actually measure different variables; in the case of flow cytometry it is cell concentration (which is affected by the replication cycle and is thus not truly exponential) and in the case of OD_750_ it is light scattering, comprising poorly-defined contributions from cell concentration, size and granularity. The true growth constant in terms of biomass dry weight is not actually provided by either method, though typically in the algal field this would be assumed to be equal to the specific growth rate estimated from optical density.

Figures 2 and 3 compare the sensitivity of microplate OD_750_ measurements to cell concentration estimation by flow cytometry. A 30 µL sample using the flow cytometer's high throughput sampler (HTS) provides greater accuracy and reproducibility than the corresponding OD_750_ measurement from a microplate reader, though with the disadvantage that, unlike OD_750_ measurements, for flow cytometry an actual sample must be removed from the well.

For *C. vulgaris* the contrast between OD_750_ and flow cytometry was particularly marked. A clear circadian rhythm was visible in the flow cytometry data (Figure 3c,d; e.g. growth plateau between 28 h–42 h) but not in the OD_750_ data (Figure 2b), again reflecting the fact that OD_750_ measurements conflate both cell size and cell concentration, whereas flow cytometry is used here just to provide the cell concentration and thus more closely tracks the replicative cycle. This is also the reason for the higher growth rates obtained from flow cytometry, which reflect the intermittent nature of cellular replication in a synchronised culture. Growth constants from these cultures were also estimated from data at 42 h onwards, but log-transformed plots of the data used in Figure 3c and Figure 3d clearly show a plateau in between the replicative cycles due to the intrinsic circadian cycle. The growth rate estimates derived from the replication cycles are lower as the culture density increases. While we have no definitive explanation for this, we note that the quality of the data (r^2^ = 0.94) is considerably higher than for the OD_750_ data (Supplementary Figures 1 and 2).

### Neither OD_750_ nor cell concentration correlates reliably to dry weight in *C. vulgaris*

Although Figure 3 demonstrates that tracking cell growth rates can be achieved more sensitively by flow cytometry than OD_750_, cell densities are not proportional to biomass dry weight unless the average cell size and buoyant density remains constant, a condition which is unlikely to be met in high-throughput growth experiments using a range of different growth conditions.

**Figure 4:**
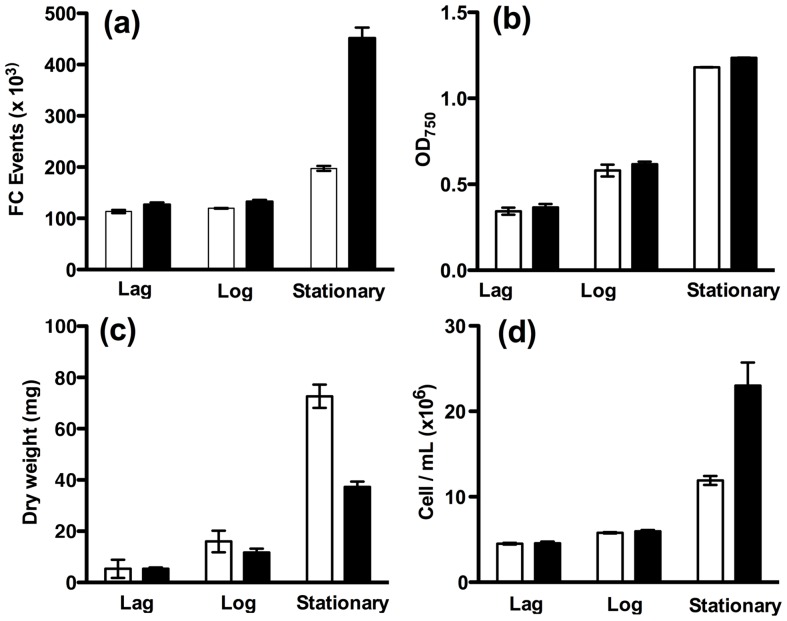
Biomass determination by dry weight, compared to OD_750_ and cell concentration (by flow cytometry and direct counting) at three points in the growth curve. Biomass production in 100*C. vulgaris* in g dry weight (g_dw_) L^−2^ day^−1^ was estimated by collecting 3×12 ml at each of 3 time points; “lag phase” (t = 0), “log phase” (after ∼10 hours of growth) and “stationary phase” (24–27 hours; immediately after growth stabilised). Filled bars signify cells grown in TAP, open bars represent cells grown in TP + 100 mM glucose. Measurements at each time point were (a) Flow cytometry (200 µL culture was diluted into 1.5 mL TP and cell concentration measured by flow cytometry) (b) OD_750_ (estimated in triplicate using 100 µL aliquots of culture diluted with 900 µL TAP or TP + 100 mM glucose) (c) Dry weight determinations (performed as described in the Methods section and (d) Microscopy (cell concentration was estimated microscopically in triplicate using a haemocytometer, from 10 µL of culture). Error bars are SEM (n = 3).

In Figure 4, flow cytometry and OD_750_ data is compared to gravimetric biomass dry weight measurements to assess the accuracy of each measurement method on actual biomass accumulation. *C. vulgaris* cultures were grown in the presence of two heterotrophic substrates; acetate (TAP) or glucose (TP + 1100 mM glucose). Samples (12 mL) were taken at three points in the culture (lag phase, log phase and stationary phase) for measurements of dry weight, OD_750_ and cell concentration (both by haemocytometer and flow cytometry).

Flow cytometry (Figure 4a) and haemocytometer measurements (Figure 4d) correlated well and indicate that after a delay, *C. vulgaris* cells grown in TAP replicate much more rapidly than cells in glucose, leading to approximately twice the cell concentration by the time stationary phase is reached. However, OD_750_ measurements (Figure 4b) show no observable difference between growth in TAP and growth in TP + glucose, with modest growth observed during the early log phase. If OD_750_ were calibrated to a typical growing culture of *C. vulgaris*, this would lead to similar biomass yields and cell densities being predicted for TAP-grown cultures and TP + glucose cultures.

In contrast, gravimetric biomass measurements (Figure 4c) show a dramatic increase in biomass for cells grown in glucose compared to those in TAP, with the final biomass yield approximately two-fold greater, in direct opposition to the cell densities observed in Figure 4a and Figure 4d. Microscopic examination confirms that this effect of glucose is correlated with a rapid increase in cell size, while replication is inhibited relative to growth in TAP. It is possible that as nitrogen becomes limiting, the presence of glucose signals to *C. vulgaris* to inhibit cell division earlier than in TAP medium and to focus on the accumulation of biomass. In this case, any attempt to use either cell concentration or OD_750_ to estimate the biomass dry weight of the populations grown on glucose would result in an underestimate.

### Pulse-width measurement can be used to estimate cell diameter and volume

Clearly, an attempt to use flow cytometry to measure biomass requires an estimation of the mass of cells in the population rather than just the cell concentration. Initially, flow cytometry was tried as a way to estimate average cell diameter [15], which is typically used by particle sizing methods to estimate biovolume by calculation. Cell diameter estimation by FC captures information concerning the population size distribution in the culture, which is missing from simple OD_750_ measurements.

**Figure 5:**
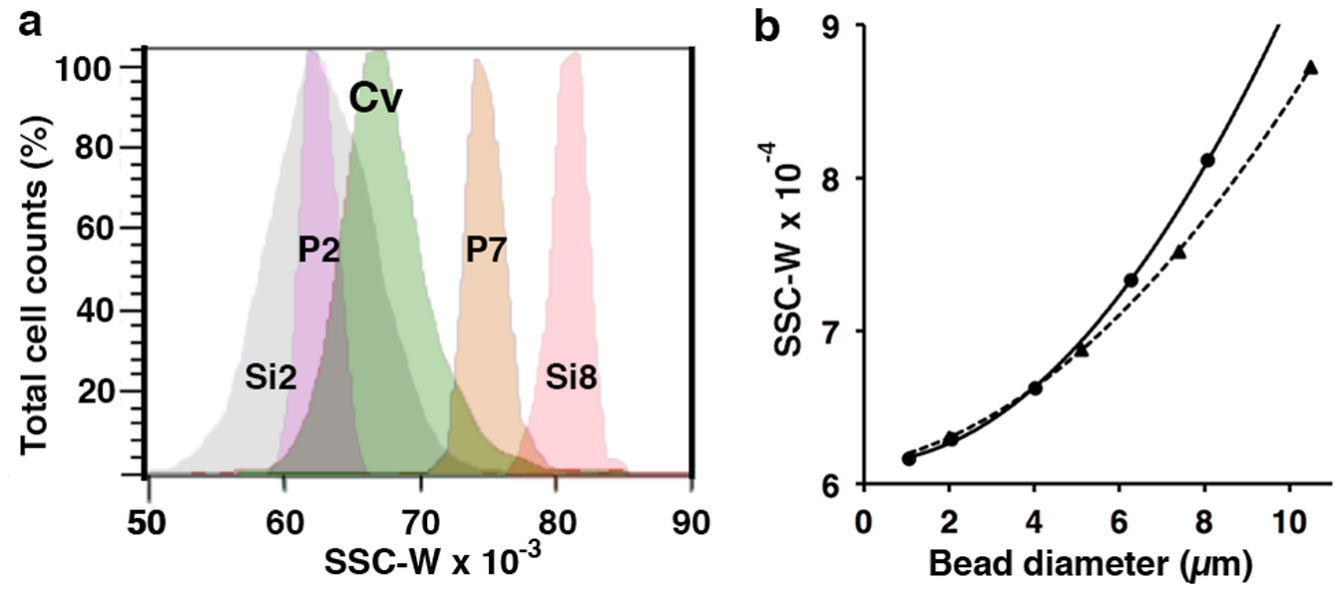
Use of flow cytometry side scatter pulse width (SSC-W) to estimate bead diameter. Mean SSC-W was estimated for a range of silica and polystyrene size marker beads. (a) Histogram plots of silica beads (Si2: 2 µm; Si8: 8 µm) and polystyrene beads (P2: 2 µm; P7: 7.4 µm) contrasted with the SSC-W profile of *Chlorella vulgaris* (Cv). (b) Calibration curves of SSC-W vs bead diameter for silica (•) and polystyrene (▴) beads.

**Figure 6:**
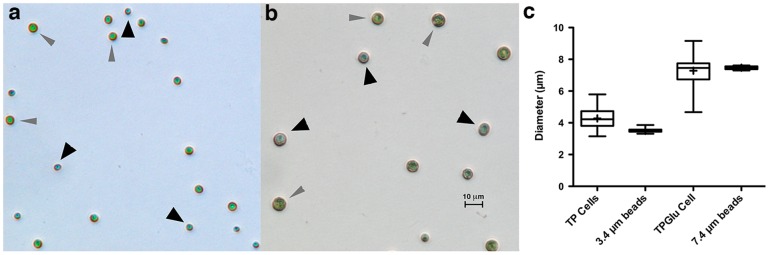
Flow cytometry pulse width measurements correlate well with cell diameter. Fluorescence activated cell sorting (FACS) of *C. vulgaris* cells was used to sort a mixture of cells and beads, according to SSC-W range. Cells and polystyrene marker beads were photographed after sorting (a) in the range SSC-W = 60000–65000 (cells grown in TP), and (b) in the range SSC-W = 70000–80000 (cells grown in TP+100 mM glucose). Representative beads (black arrows) and cells (grey arrows) marked are indicated. (c) Diameters were measured microscopically using IMARIS software for ∼100 cells or beads after sorting and the mean, standard deviation and range was plotted for each population of cells and the marker beads.

Figure 5 shows that size calibration beads can be used to estimate the cell diameter of a population from side scatter pulse width (SSC-W) flow cytometry data, while figure 6 shows that this correlates well with cell diameter as estimated from light microscopy. Algal cells have a refractive index (RI) around 1.41–1.43 [19], [20] whereas the RI of polystyrene beads is around 1.9. This discrepancy is critically important for the use of forward scatter to estimate cell size [21] using Rayleigh-Gans theory, which is a theoretical approximation used for calculating light scattering and which relies heavily upon refractive index. However, RI is less important for pulse width data because the measured variable is not the intensity (height, area) of the scattered light but the pulse width (time) which is a near linear function of cell diameter (given a constant flow rate through the flow cell), though as cells become smaller than the width of the laser beam, a nonlinearity appears which Sharpless and Melamed [16] demonstrated can be modelled relatively simply. To check the effect of refractive index, we compared pulse width data for silica microspheres (RI 1.4) with that for polystyrene beads, which are more commonly used in flow cytometry laboratories. As shown in Figure 5, there was little difference in pulse width data between the bead types (though a discrepancy does begin to appear at higher bead diameters). This observation agrees with the study by Sharpless et al. [22] demonstrating that unlike intensity measurements, pulse width data is less affected by refractive index, though silica beads are still preferred due to the closer correspondence with the RI of cells.

Beads have a defined diameter and a hard edge, whereas algal cells have a transparent cell wall and a complex internal structure, and dynamic light scattering experiments have shown that algal cells cannot be accurately modelled simply as spheres using Mie scattering theory [23], [24]. It was therefore possible that cell diameter (as estimated microscopically) might differ from cell diameter measured by laser scattering pulse width. To confirm that cells and beads sorted in a given SSC-W (side scatter width) bin were in fact similar in diameter, a fluorescence activated cell sorter (FACS) was used to sort a combination of cells and calibration beads at a series of SSC-W bins, and microscopy was used to confirm that the beads co-sorted with cells of a similar diameter (Figure 6), at least in the case of *C. vulgaris.*

Cells purified from the 3.4 µm bead sort are slightly larger than 3.4 µm on average, possibly because of overspill from the main *C. vulgaris* peak, as there are few cells in the 3 µm range in the *C. vulgaris* population. However, the correlation is good, at least for *C. vulgaris*, which is a spherical alga. It is expected that non-spherical cells such as diatoms will align with the sheath fluid flow along their long axis and that the relationship between cell diameter and bead diameter will not be identical, but as the deviation should be linear, a simple correction factor should be adequate. Further work will be necessary to confirm whether this assumption holds.

### Relationship between biovolume, buoyant density and dry weight density

It is possible to calculate average (wet) biovolume from average cell diameter, while average buoyant cell density can be measured by density gradient centrifugation; consequently, wet biomass can be calculated. If there were a simple relationship between wet and dry biomass, the biomass dry weight could be estimated from the wet weight. Unfortunately this is not the case. The dry matter content of cells varies greatly both within a population and between populations, not only for algae but for planktonic species in general [4], and empirical calibration for each species is necessary to account for this.

Since such calculations of biomass are derived in the first place from FC estimates of cell diameter, we explored the direct empirical relationship between the measurement of FC pulse width parameter (used to estimate cell diameter) and the subsequent measurement of dry weight biomass per cell. While still requiring an empirical correlation to dry weight for each species, this avoids the need to calculate any intermediate parameters.

The FC measurement includes information related to both cell size and cell concentration. An independent measurement of dry weight biomass of the same sample enables calculation of the average dry weight per cell. The relationship between these measurements is plotted for several populations under a range of growth conditions which have, as a result, different average cell sizes.

**Figure 7:**
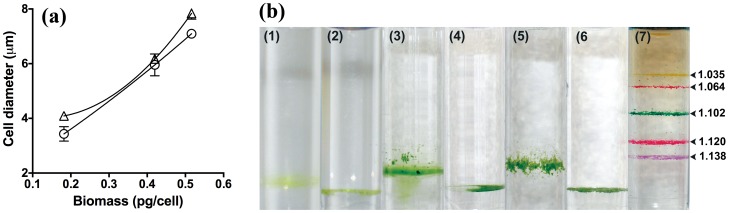
Partial dehydration reduces density differences between cell populations. *C. vulgaris* biomass was measured during lag phase, log phase and stationary phase in TP with 100 mM glucose. (a) Flow cytometry was used to estimate average cell diameter using SSC-W measurements and plotted vs average dry weight per cell. Flow cytometry was performed on cells diluted into TP growth medium (▵) or hypertonic media (1 M NaCl in TP) (▵) and allowed to dehydrate for 10min. The use of NaCl improves the linearity of the relationship between biovolume and biomass dry weight. All measurements were conducted in triplicate; error bars represent the standard error of the mean. (b) Percoll density gradient centrifugation demonstrates a lower, more uniform range of cell densities when measured in medium containing 1 M NaCl. Tubes are labelled as follows: (1) *C. vulgaris* in TP. (2) *C. vulgaris* + 1 M NaCl. (3) *C. vulgaris* in TAP. (4) *C. vulgaris* in TAP + 1 M NaCl 1 M. (5) *C. vulgaris* in TP + glucose (100 mM). (6) *C. vulgaris* in TP + glucose (100 mM) + 1 M NaCl. (7) Standard density marker beads (g.mL^−1^) with densities indicated.

Figure 7a shows that for fully hydrated cells assayed in TP medium, the empirical relationship between cell diameter and biomass dry weight per cell is curvilinear, so that, as expected, biovolume cannot be converted to dry mass using a simple (linear) conversion factor. This illustrates that on average, the water content of a small cell is different to that of a large cell; however no simple volumetric equation was found which fitted the empirical data effectively and it was decided to rely upon calibration curves rather than trying to predict the relationship *ab initio*.

### Hypertonic conditions linearise the relationship between cell diameter and cellular dry weight

**Figure 8:**
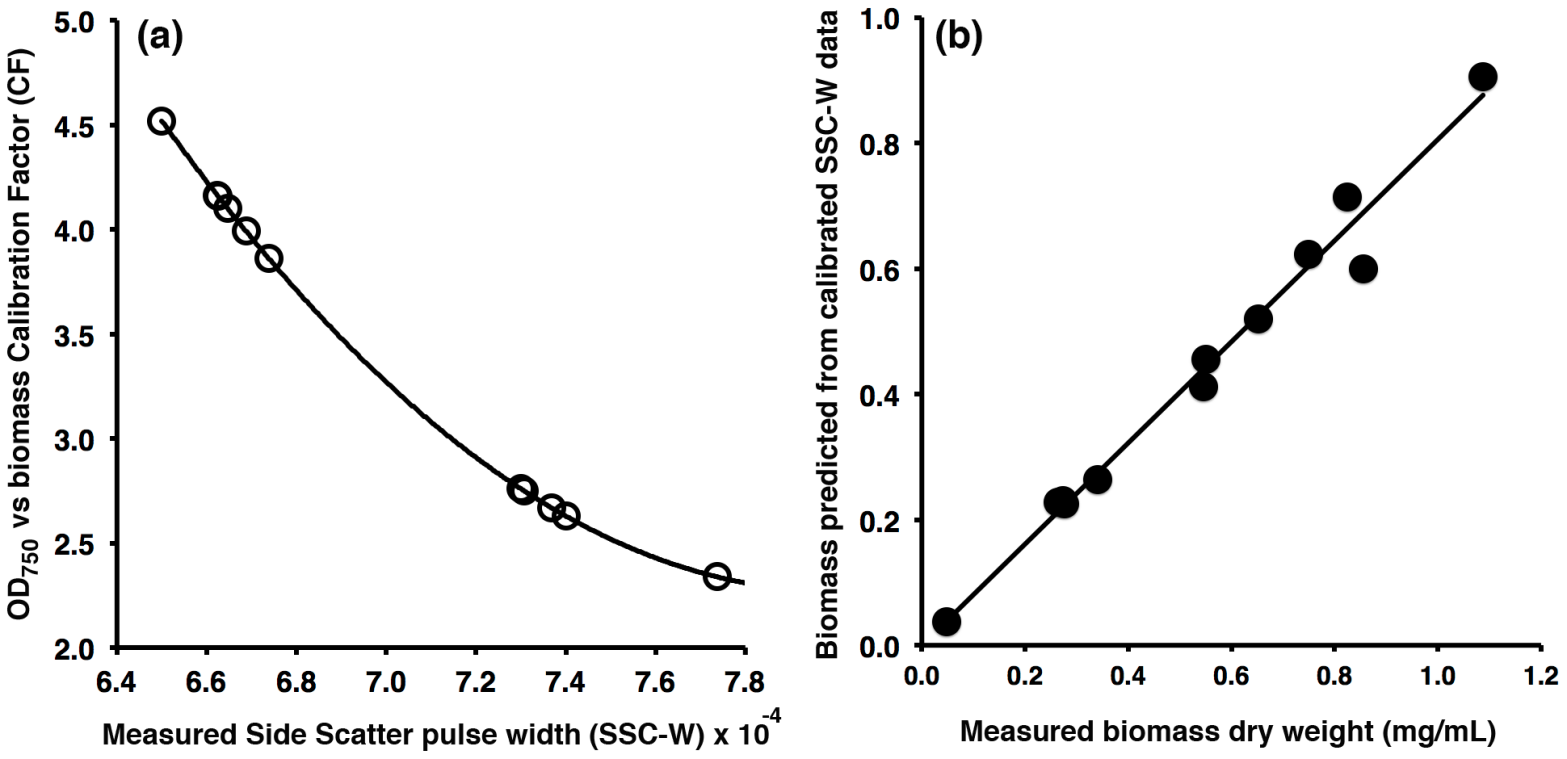
OD_750_ measurements can be corrected using flow cytometry to yield reliable biomass estimates. (a) OD_750_ calibration constants (i.e. the slope of OD_750_ vs dry weight biomass) were measured for a range of *C. vulgaris* cultures at different growth stages (with different average cell diameter). These values were plotted against flow cytometry (peak SSC-W) measurements taken on the same cultures. (b) Using this calibration curve, biomass dry weight was then predicted for a different set of previous cultures for which both biomass and OD_750_ were known. Comparison of the predicted biomass and measured biomass demonstrates a linear relationship (r^2^ = 0.981), suggesting the use of FC measurements to correct OD_750_ measurements enables a linear relationship to be established between OD_750_ and biomass dry weight regardless of the cell morphology or culture stage.

If the empirical relationship between SSC-W and dry biomass per cell were linear, a simple conversion factor could be used rather than a calibration curve. We reasoned that if the curvilinear relationship in Figure 8 were due to variable water content, the use of hypertonic flow cytometry conditions would lessen this curvature, as cells will shrink until the intracellular water content reaches a minimum which reflects the non-aqueous cell components plus a characteristic amount of tightly bound water [25]. Under these partially dehydrating conditions, the cellular volume should be much more closely related to the biomass dry weight, since the density difference between different cell populations has been equalised (or at least, made more similar). Ethanol, methanol and NaCl were explored as dehydrating agents. Remarkably, in such partially dehydrated cells the resultant cell diameter estimates were almost linearly related to the measured dry weight (Figure 7a – cells assayed after 10min exposure to 1 M NaCl). Percoll density gradients carried out in these dehydrating conditions also showed a tight uniform buoyant density even for morphologically different *C. vulgaris* populations (Figure 7b; *C. vulgaris* grown in TAP vs. TP + 1100 mM glucose) suggesting that differences in buoyant density are also related partly to a variable water content.

Although for simplicity the linear relationship derived from cellular dehydration is useful, even a non-linear calibration curve can be used to estimate biomass accurately from biovolume as long as the average cell diameter can be estimated. Furthermore, although the relationship between cell diameter and biomass per cell (Figure 7a) is presented here for theoretical reasons, a direct plot of the primary measured FC parameter (SSC-W) under hypertonic conditions vs. dry biomass (mg/mL) provides a more direct relationship between the observed SSC-W data and the biomass dry weight. Therefore, while determination of the cell diameter from SSC-W data is desirable information in its own right, it is not actually necessary to estimate the biomass; the SSC-W can be plotted directly against biomass dry weight, avoiding both a data processing step and the bead-based calibration.

### Use of flow cytometry to correct optical density measurements

Finally, the use of intermittent flow cytometry measurements to correct OD_750_ measurements was explored, for use when OD_750_ is the preferred monitoring method. If calibration factors between OD_750_ and biomass (as illustrated in Figure 1c) are known for populations having different average cell sizes, then flow cytometry can potentially be used to choose the correct calibration value for a given OD_750_ measurement.

The OD_750_-to-biomass (dry weight) calibration factor (CF) was measured for several cultures at different growth stages, similarly to Figure 1c. At the same time, the average SSC-W value for the culture was recorded using flow cytometry. The relationship between these variables (Figure 8a) shows that by measuring the average SSC-W of a culture, the correct CF can be chosen to reliably convert an OD_750_ measurement to biomass dry weight. An illustration of this is provided in Figure 8b where the OD_750_ in conjunction with SSC-W was used to predict biomass dry weight for a set of independent cultures, with good correlation (r^2^ = 0.98).

Although the FC measurement itself can be used to determine biomass dry weight for the sample, this combined approach may be advantageous where a set of cultures, monitored by OD_750_, are known to be changing in a coordinated way, or where the change in the population size distribution is slow relative to the change in OD_750_ (for example, during a dynamic mixing situation). In such cases a single FC measurement can be used to select the correct CF for subsequent OD_750_-to-biomass dry weight estimates for a whole set of samples or measurement times.

The relative advantages of optical density vs. flow cytometry will depend on the experimental design, but a combination will often be advantageous. Where the cell concentration of a sample is high enough for accurate optical density measurements, but a range of growth conditions are being examined, flow cytometry can improve the accuracy of biomass estimation by OD_750_. Conversely, where cell concentration is low and sensitivity is more of an issue, a direct biomass estimation by flow cytometry under dehydrating conditions can be made. Finally, measuring just the SSC-W requires fewer events than required for an accurate estimate of cell density and is subject to fewer measurement errors (e.g. cell settling) so that a much smaller sample size (a few hundred cells) can be taken.

## Discussion

Dry biomass yield, composition and cell concentration are critical metrics for algal biotechnology applications, including algal biofuel systems. The ability to accurately and repeatedly measure mass and composition under a broad variety of growth conditions is especially desirable for high throughput, small volume assays aimed at optimising growth conditions, light regimes and experimental manipulations. Although the measurement of biomass dry weight conveys no information about biomass composition, it is a widely used indicator of system efficiency, as it effectively records the conversion of macroelements (C, N, O, P) into organic biomass. Unfortunately, true dry weight measurements are not practical for small volumes of culture.

Optical density is one of the simplest methods of measuring culture growth, though not very sensitive at low cell densities. As a standalone technique, accurate calibration for biomass estimation is only possible if the cell morphology, size and density do not change during growth. This is true for some species including *Chlamydomonas reinhardtii*, but does not apply to others such as *Chlorella vulgaris*, as illustrated here. The dramatic increase in cell size and dry weight when *Chlorella sp.* are grown on glucose was noted as early as 1936 [26], [27]. Griffiths [28], [29] reported that *C. vulgaris* (211/11n; also known as “Emerson strain”) would not proliferate on glucose in the dark, but that “giant cells” developed when glucose was available but limited light led to the cessation of cell division.

### Flow cytometry for biomass determination

Flow cytometry allows the measurement of particle diameter and hence biovolume. Like other techniques which measure particle size (DLS, Coulter techniques) estimation of wet mass or buoyant mass is not difficult, but conversion of wet mass to dry weight requires estimating the average mass fraction which is often undefined for a given sample. Flow cytometry is a versatile technique which is not only widely available but has a long history of use in phycology. Whereas FSC-A and SSC-A measurements are routine, SSC-W measurements are not usually collected during algal flow cytometry, and have not, to our knowledge been previously used for algal biomass estimation. Despite this, the collection of SSC-W data is within the capacity of most flow cytometers and involves no extra work if flow cytometry is already being used for other purposes. In modern flow cytometers, a photodiode instead of a photomultiplier is used for measuring forward scatter. As photodiodes are less sensitive and have a nonlinear response, FSC-W is considered less suitable than SSC-W for measurements of cell dimensions. We note that pulse width data from chlorophyll fluorescence could also be used if the chloroplast is a physically dominant organelle in the cell (e.g. in *Chlamydomonas*), but not where it is a smaller component (e.g. in many diatoms).

The results reported here show that flow cytometry can be used as an alternative or complementary method to accurately estimate true biomass dry weight in cultures which have small volumes (<100 µL) and low cell densities (fewer than 1000 cells µL^−1^) such as high throughput screening assays. The primary requirement is to establish the empirical relationship between an optical parameter (flow cytometry) and the biomass dry weight, as shown in Figure 7.

### The relationship between cell size and biomass dry weight

The major contributor (∼50%) to cellular dry biomass is carbon. As carbon corresponds to fixed CO_2_, and the next most abundant element is oxygen (∼35%), carbon is usually also the element of most interest. The relationship between cellular carbon and cell volume is complex and is the subject of a large literature in oceanography. Key problems include the discrimination of photosynthetic cells from inorganic material, and estimation of total organic carbon content. In 1999, Stramski proposed that the polarised components of refractive index could be used to independently estimate the carbon and chlorophyll contents of algal cells. Building on this approach, Mahlmann et al. [30] used Mach-Zehnder double-beam interference microscopic interferometry to estimate the carbon content of individual cyanobacterial cells, and Popescu et al. [31] used a similar approach to estimate dry weight in mammalian cells in tissue culture.

Although individual cell microscopy techniques are not practical for high throughput analysis, flow cytometry could be used, with suitable interference optics, to independently measure cellular carbon content which, in conjunction with biovolume measurements, would enhance the accuracy of biomass estimation using flow cytometry alone. Past studies employing flow cytometry to estimate biomass [19], [32] utilised forward scatter to estimate cell diameter and corrections for the chlorophyll fluorescence signal to estimate total chlorophyll; the accuracy of these approaches may be improved by employing side scatter pulse width. As flow cytometry is widely employed in ecological studies, our methods could also assist with accurately estimating biomass in aqueous ecosystems.

Like *C. reinhardtii* and *C. vulgaris*, many of the microalgal species used in algaculture research and development are spherical in shape. In future, it will also be interesting to see the extent to which cell shape influences biovolume estimation by flow cytometry, and the range of cell shapes that can be measured in this way. The use of lipid- vs. starch-packed cells, the silicate frustules of diatoms and the inclusion of cells containing air vacuoles may lead to a wider range of cell densities than measured here and more broadly test the accuracy of the present method. A thorough investigation of the variability in cell density and the relationship between buoyant mass, dry weight and cellular composition is therefore clearly important, though outside the scope of the present work.

One important source of variation in cell density is the cellular water content. Cytoplasmic osmolality depends on the ratio between biopolymers and the small molecules from which they are composed. The cleavage of polymers such as starch to simple monomers results in an increase in the cellular ion concentration and cellular hydration varies accordingly. Also, because fresh water algae typically grow in media of much lower osmolality than the cytoplasmic contents, failure of the cell to maintain membrane and cell wall integrity tends to result in hypotonic shock and expansion in cellular volume. Changes in cellular hydration have relatively little impact on wet density because the density of hydrated biopolymers is not greatly different to that of water, but there is a large effect on biomass dry weight.

### Cellular water content under dehydrating conditions

We found that dehydration using salt solutions reduced the buoyant density variations in these algal species and helped to linearise the relationship between flow cytometry measurements and biomass dry weight. The issue of cellular hydration has been studied in the cryopreservation of microalgae, where formation of intracellular ice crystals is minimised by removal of as much water as possible. Typically, dehydrating agents such as methanol are employed to achieve this, but there is often a balance between osmotically driven water flow out of the cell and concentration driven influx of the cryoprotective agent. Tanaka et al. [25] used video microscopy to measure the rates of transfer of water and cryoprotective agents across the cell wall of *Chlorococcum texanum* and showed that exposure to hyperosmotic media caused an immediate shrinkage of the cell, typically ∼45%, to a minimum equilibrium volume where the remaining water is considered to be bound by macromolecules. Notably in our experiments the greatest effect of dehydration was upon the largest cells, with relatively little change seen in small cells, where it is expected that the cellular contents are dictated by the minimal size of critical cellular components such as the nucleus, cytoskeleton, chloroplast and mitochondria. The degree of shrinkage seen with algal species will probably vary with the strength of the cell wall. Such dehydration may create a more uniform relationship between cell size and dry weight, but clearly it does not produce a constant dry weight mass fraction in relation to volume. The effectively linear relationship we observed between cell diameter and cellular biomass dry weight was unexpected, even given the complex literature in this field.

### High-throughput continuous monitoring of algal cultures

The advantages of optical density measurements are simplicity, economy and the potential for continuous online monitoring. These advantages make OD_750_ the logical choice for monitoring culture growth when an accurate biomass yield is not the primary variable of interest, or where an empirically calculated calibration between OD_750_ and biomass remains valid. If these conditions do not hold, flow cytometry can provide a convenient and accurate assay. It has the added advantage of being a sensitive, flexible technique with the potential for simultaneous measurement of many other cellular variables.

Another advantage of flow cytometry is the ability to discriminate between cell concentration and cell size during the measurement of biomass. Flow cytometry profiles have long been used to track the replicative cycle of algal populations [33]–[38]; given the propensity of some algae to maintain strong circadian rhythms, this conveys critical information concerning the behavioural state of the algal cell population, in particular whether the algae are replicating rapidly or simply growing in average size per cell. The results obtained here with *Chlorella vulgaris* illustrate this point, and suggest that flow cytometry can provide a detailed analysis of growth regimes enabling a rational, empirically-based understanding of biomass accumulation in relation to growth conditions.

The applicability of our methods beyond *C. vulgaris* and *C. reinhardtii* hinges on the ability to accurately correlate flow cytometry pulse width (a proxy for cell diameter) and density. For some cell types, for example diatoms and algae with small numbers of discrete chloroplasts, the side scatter pulse width may not vary as much with cell diameter and the correlation with biomass may not be so informative. Although forward scatter has also been used to estimate cell size [32], [39], [40] pulse width is a more reliable metric [15], [41] and reduces the importance of obtaining beads with a matching refractive index [21], though this is still desirable. In fact, according to Sharpless and Melamed [16] once an instrument has been calibrated for bead size, peak width data provides thereafter an absolute measure of cell diameter, so that recalibration in each experiment should be unnecessary, which we have found to be the case.

## Conclusions

In summary, flow cytometry can be used to accurately estimate biomass dry weight in *C. vulgaris* and *C. reinhardtii* cultures containing very few algal cells. These data provide confidence in miniaturised assays where biomass dry weight is an important variable, or where frequent and rapid estimates of dry mass are required. At the same time, where the convenience of optical density is crucial, accuracy in biomass estimation can be usefully improved by supplementary flow cytometry data.

## Supporting Information

Figure S1 **Growth curves from** **Figure 2** **presented as the natural logarithm.**
(PDF)

Figure S2 **Growth curves from** **Figure 3** **presented as the natural logarithm.**
(PDF)
