# Supplementary material for: Flow Cytometry Pulse Width Data Enables Rapid and Sensitive Estimation of Biomass Dry Weight in the Microalgae Chlamydomonas reinhardtii and Chlorella vulgaris
Source: PLoS One. 2014 May 15;9(5):e97269. doi: 10.1371/journal.pone.0097269 (PMC4022489; doi:10.1371/journal.pone.0097269)
Supplement: Figure S1 — Growth curves from Figure 2 presented as the natural logarithm. (PDF) [file pone.0097269.s001.pdf]

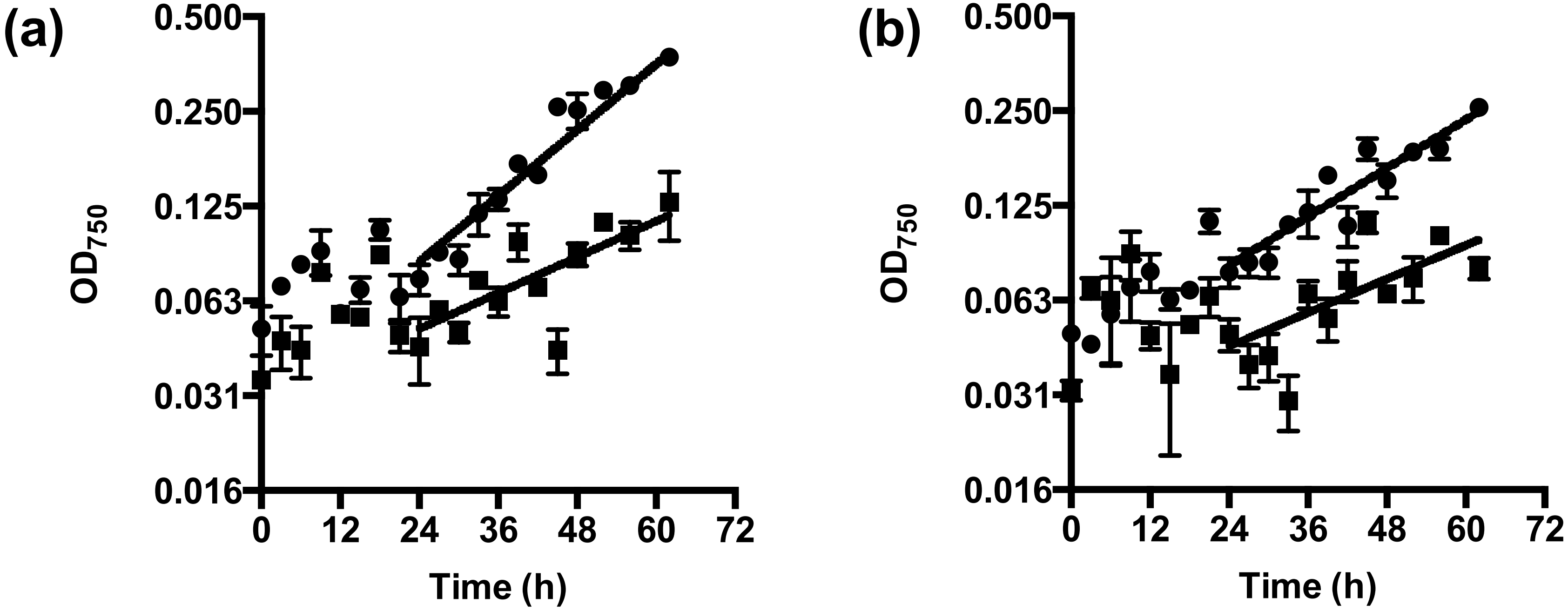

Supplementary Figure S1: Log_2 transformed growth curves from optical density at 750nm. Data is the same as in Figure 2. Growth curves monitored in 96 well plates by OD_{750} in a plate reader (effective path length ~0.2cm) at different initial cell densities. (a) *Chlamydomonas reinhardtii* in TAP at initial cell density of 10^6 cells/mL (o) and at initial cell density of $2.5x10^5$ cells/mL (●). (b) *Chlorella vulgaris* in TAP at initial cell density of 10^6 cells/mL (☐) and at initial cell density of $2.5x10^5$ cells/mL (■). Error bars are SEM (n=3). Slopes of the fitted lines were used to derive specific growth constants. Slopes were measured from 24h onwards to avoid the lag phase period.
