# Supplementary material for: Flow Cytometry Pulse Width Data Enables Rapid and Sensitive Estimation of Biomass Dry Weight in the Microalgae Chlamydomonas reinhardtii and Chlorella vulgaris
Source: PLoS One. 2014 May 15;9(5):e97269. doi: 10.1371/journal.pone.0097269 (PMC4022489; doi:10.1371/journal.pone.0097269)
Supplement: Figure S2 — Growth curves from Figure 3 presented as the natural logarithm. (PDF) [file pone.0097269.s002.pdf]

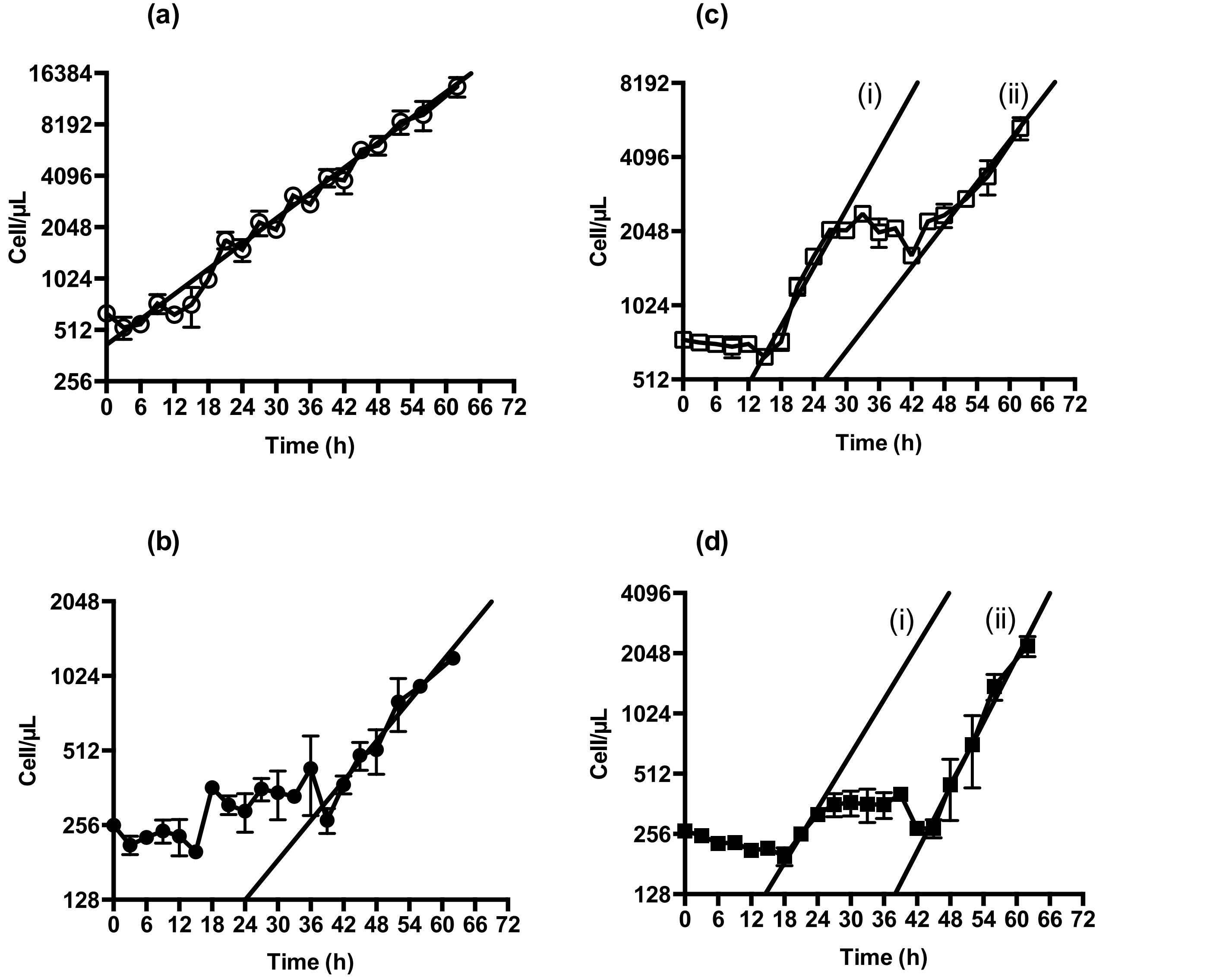

Supplementary Figure S2: Log_2 transformed growth curves from flow cytometry. Data is the same as in Figure 3. Cell density (cells/µL) was estimated from flow cytometry events, calibrated using CountBright™ Absolute Counting Beads (Invitrogen™). Error bars are SEM (n=3). (a) *Chlamydomonas reinhardtii* strain 137c at initial cell density of 10^6 cells/mL (O). (b) *C. reinhardtii* strain 137c at initial cell density of 2.5×10^5 cells/mL (●). (c) *Chlorella vulgaris* at initial cell density of 10^6 cells/mL (□). (d) *C. vulgaris* at initial cell density of 2.5×10^5 cells/mL (■). Slopes of the fitted lines were used to derive specific growth constants. In the case of *C. vulgaris*, slopes were measured during both the first (i) and second (ii) rounds of cell division.
